# Atrial natriuretic peptide protects against bleomycin-induced pulmonary fibrosis via vascular endothelial cells in mice: ANP for pulmonary fibrosis

**DOI:** 10.1186/s12931-016-0492-7

**Published:** 2017-01-03

**Authors:** Atsuko Okamoto, Takashi Nojiri, Kazuhisa Konishi, Takeshi Tokudome, Koichi Miura, Hiroshi Hosoda, Jun Hino, Mikiya Miyazato, Yohkoh Kyomoto, Kazuhisa Asai, Kazuto Hirata, Kenji Kangawa

**Affiliations:** 1 Department of Biochemistry, National Cerebral and Cardiovascular Center Research Institute, 5-7-1, Fujishirodai, Suita-City, Osaka 565-8565 Japan; 2 Department of Respiratory Medicine, Osaka City University Graduate School of Medicine, 1-4-3, Asahi-machi, Abeno-ku, Osaka-City, Osaka 545-8585 Japan; 3 Department of Regenerative Medicine and Tissue Engineering, National Cerebral and Cardiovascular Center Research Institute, Suita-City, Osaka Japan

**Keywords:** Atrial natriuretic peptide, Pulmonary fibrosis, Bleomycin, Vascular endothelial cell, Transforming growth factor-β

## Abstract

**Background:**

Pulmonary fibrosis is a life-threatening disease characterized by progressive dyspnea and worsening pulmonary function. Atrial natriuretic peptide (ANP), a heart-derived secretory peptide used clinically in Japan for the treatment of acute heart failure, exerts a wide range of protective effects on various organs, including the heart, blood vessels, kidneys, and lungs. Its therapeutic properties are characterized by anti-inflammatory and anti-fibrotic activities mediated by the guanylyl cyclase-A (GC-A) receptor. We hypothesized that ANP would have anti-fibrotic and anti-inflammatory effects on bleomycin (BLM)-induced pulmonary fibrosis in mice.

**Methods:**

Mice were divided into three groups: normal control, BLM with vehicle, and BLM with ANP. ANP (0.5 μg/kg/min via osmotic-pump, subcutaneously) or vehicle administration was started before BLM administration (1 mg/kg) and continued until the mice were sacrificed. At 7 or 21 days after BLM administration, fibrotic changes and infiltration of inflammatory cells in the lungs were assessed based on histological findings and analysis of bronchoalveolar lavage fluid. In addition, fibrosis and inflammation induced by BLM were evaluated in vascular endothelium-specific *GC-A* overexpressed mice. Finally, attenuation of transforming growth factor-β (TGF-β) signaling by ANP was studied using immortalized mouse endothelial cells stably expressing GC-A receptor.

**Results:**

ANP significantly decreased lung fibrotic area and infiltration of inflammatory cells in lungs after BLM administration. Furthermore, similar effects of ANP were observed in vascular endothelium–specific *GC-A* overexpressed mice. In cultured mouse endothelial cells, ANP reduced phosphorylation of Smad2 after TGF-β stimulation.

**Conclusions:**

ANP exerts protective effects on BLM-induced pulmonary fibrosis via vascular endothelial cells.

## Background

Pulmonary fibrosis is a life-threatening disease characterized by progressive dyspnea and worsening pulmonary function [1]. Although the pathologic processes that cause disease progression are not fully understood, the common pathological features of pulmonary fibrosis are infiltration by inflammatory cells, including activated macrophages [2]. No effective therapeutic strategies for this condition have been established; therefore, development of effective treatment options is desirable.

Bleomycin (BLM)-induced pulmonary fibrosis is the rodent model most commonly used to study idiopathic pulmonary fibrosis [3]. Administration of BLM causes epithelial injury, followed by neutrophil-dominant and lymphocyte-dominant inflammation that leads to fibrosis [4]. Vascular endothelial cells are a major target of BLM-induced pulmonary fibrosis [4, 5].

Atrial natriuretic peptide (ANP) is a heart-derived secretory peptide that mediates a wide range of biological functions including diuresis, natriuresis, vasorelaxation, and inhibition of the renin–angiotensin–aldosterone system. These effects are mediated by specific binding of ANP to the guanylyl cyclase-A (GC-A) receptor [6, 7]. The GC-A receptor is predominantly expressed in the heart and vascular endothelium, indicating that the cardiovascular system is the main target for treatments using ANP [6–8]. ANP exerts protective effects in a wide range of organs, including the heart, blood vessels, kidneys, and lungs, in which it exhibits both anti-inflammatory and anti-fibrotic activities [6–10]. Previous studies showed that ANP administration has beneficial effects on acute lung injury in patients requiring mechanical ventilation [11] or who experience postoperative respiratory and cardiovascular complications following lung cancer surgery [12, 13]. Recently, we reported that ANP exerts a protective effect against lipopolysaccharide-induced acute lung injury in mice by suppressing vascular E-selectin expression [10]. On the basis of these studies, we hypothesized that ANP may reduce BLM-induced pulmonary fibrosis, in particular via vascular endothelial cells. In this study, we investigated the protective effects of ANP on BLM-induced pulmonary fibrosis using vascular endothelium–specific *GC-A* overexpressed mice.

## Methods

### Animal studies

C57BL/6 N mice (male, 7 weeks old, weighing 21–23 g each) were purchased from Japan SLC (Shizuoka, Japan). We previously established the overexpressed mice for Tie2-Cre-inducible overexpression of *GC-A*, which is termed the endothelium-specific *GC-A* overexpressed mice in this study. We previously confirmed that Tie2-Cre-*GC-A* overexpression mice showed GC-A protein of vascular endothelial cells in the lung was upregulated compared to wild type mice [14]. Animals were maintained at a controlled temperature of 24 °C ± 1 °C under a 12:12 h light–dark cycle, and were fed a standard diet. Water was freely available. All experimental protocols described herein were approved by the Animal Care Ethics Committee of the National Cerebral and Cardiovascular Center Research Institute, Japan.

### BLM administration and ANP treatment

The mice were anesthetized with 3% isoflurane delivered in a box, and BLM (1 mg/kg, Nippon Kayaku Co, Tokyo, Japan) in 80 μl of saline was administered via oropharyngeal aspiration as previously described [15]; an identical volume of sterile saline was administered to normal control mice. ANP (0.5 μg/kg/min, Peptide Institute Inc, Osaka, Japan) or vehicle was subcutaneously infused via an osmotic mini-pump (Alzet Model 2004, Duret Corporation, Cupertino, CA, USA), and the pumps were implanted 72 h before BLM administration, as previously described [10, 14, 15]; the infusion continued until the mice were euthanized. Mice were divided into three groups: normal control mice, BLM-treated mice receiving ANP, and BLM-treated mice receiving vehicle (*n* = 20 in each group).

### Experimental design

On day 7 after BLM administration, mice were assessed by measuring cell counts in bronchoalveolar lavage (BAL) fluid, as described below, and immunostaining. The remainder of the mice were euthanized for histological and gene expression analysis of the lung on day 21 after BLM administration. Vascular endothelium–specific *GC-A* overexpressed mice and WT littermates were also subjected to bleomycin inhalation, and then sacrificed at 21 days after BLM administration. The left lung was fixed by intratracheal instillation of 4% paraformaldehyde for 7 days, and subsequently embedded in paraffin. Paraffin sections were stained with hematoxylin–eosin and Masson trichrome (MT).

### Quantitative evaluation of lung fibrosis

Lung sections were stained with Masson trichrome, and then each slide was scanned completely in a zigzag fashion, and the percentage of fibrotic area in the whole lung field was assessed. Brightfield images of Masson trichrome-stained slides were acquired on an FSX100 system (Olympus, Tokyo, Japan) and the fibrotic area (expressed as a percentage of the whole lung field) was analyzed by using CellSens Dimension software version 1.6 (Olympus).

### BAL fluid analysis

BAL fluid was collected and assessed as previously described [15].

### Immunostaining of lung

For Mac-3 staining, tissue sections were deparaffinized, and endogenous peroxidase was blocked with 3% H_2_O_2_ for 30 min. After each step, the tissue sections were rinsed twice in phosphate-buffered saline (PBS) for 5 min. The deparaffinized tissue sections were incubated with Protein Block (DakoCytomation, Glostrup, Denmark) for 15 min. The rat anti-mouse Mac-3 antibody was diluted in an antibody diluent buffer (dilution 1:500; BioLegend, San Diego, CA, USA) and applied overnight at 4 °C. After incubation with primary antibodies, the slides were incubated with biotinylated rabbit anti-rat IgG for 60 min, followed by incubation with peroxidase-conjugated avidin–biotin complex (Vectastain ABC kit; Vector Laboratories, Burlingame, CA, USA) for 30 min. Antigen–antibody complexes were visualized with 0.5% diaminobenzidine (DakoCytomation) and 0.3% hydrogen peroxide, and then counterstained with hematoxylin.

### Gene expression analysis

Total RNA from lung was homogenized in guanidium-phenol-chloroform and isolated using the RNeasy mini kit (Qiagen, Hilden, Germany). The RNA was then reverse-transcribed into cDNA using a QuantiTect Reverse Transcription kit (Qiagen). Quantitative PCR assays were conducted in a 96-well plate using SYBR Premix Ex Taq (Takara, Siga, Japan) on a Light Cycler 480 System II (Roche Applied Science, Indianapolis, IN, USA). Primer sequences are provided in Table 1. PCR settings were as follows: initial denaturation for 30 s at 95 °C, followed by 38 cycles of 5 s at 95 °C and 20 s at 57 °C (interleukin [IL]-6); 5 s at 95 °C, 10 s at 56 °C, and 15 s at 72 °C (IL-1β); 5 s at 95 °C and 20 s at 60 °C (basic fibroblast growth factor [bFGF], transforming growth factor-β [TGF-β], connective tissue growth factor [CTGF], and 36B4); or 5 s at 95 °C and 20 s at 58 °C (monocyte chemoattractant protein-1 [MCP-1], collagen 1A, and tissue inhibitor of metalloproteinases type1 [TIMP1]). Melting curve analysis was conducted with temperature increasing from 72 to 98 °C. Gene expression levels were normalized against corresponding levels of the housekeeping gene 36B4.

**Table 1:** Sequence of primers used in the study

Gene	Forward (5′-3′)	Reverse (5′-3′)
*IL-1β*	AGCACCTTCTTTCCCTTCATCTTTG	GAGGTGGAGAGCTTTCAGTTCATAT
*IL-6*	CCAGTTGCCTTCTTGGGACTGATG	GTAATTAAGCCTCCGACTTGTGAAG
*MCP-1*	GCAGGTGTCCCAAAGAAGCTGTAGT	CAGAAGTGCTTGAGGTGGTTGTGGA
*bFGF*	GCTCTACTGCAAGAACGGCGGCTTC	ACACACTTAGAAGCCAGCAGCCGTC
*Collagen 1A*	AGTAACGTCGTGCCTAGCAACATGC	GAATACTGAGCAGCAAAGTTCCCAG
*TIMP-1*	ATCATCGAGACCACCTTATACCAGC	TGCAGGCAGTGATGTGCAAATTTCC
*TGF-β*	CAACTACTGCTTCAGCTCCACAGAG	CAAGGACCTTGCTGTACTGTGTGTC
*CTGF*	CAAGTTTGAGCTTTCTGGCTGCACCAG	GGACAGTTGTAATGGCAGGCACAGG
*36B4*	TCATTGTGGGAGCAGACAATGTGGG	AGGTCCTCCTTGGTGAACACAAAGC

### Cell culture analysis of mouse immortalized endothelial cells

SVEC, a mouse immortalized endothelial cell line, was obtained from Y. Takuwa (Kanazawa University). SVEC cells were cultured in Dulbecco's Modified Eagle Medium (DMEM) supplemented with 10% fetal calf serum (FCS). SVEC cells stably expressing GC-A-FLAG (SVEC/GC-A) were established using ecotropic retrovirus expressing the tagged protein. The details of the methodology will be described elsewhere (K. Miura et al., manuscript in revision). The SVEC/GC-A cells were treated with or without TGF-β (1 ng/ml) and/or ANP (0.1 μM) for 30 min for western blot analysis, and for 4 h for gene expression analysis.

### Western blot analysis

Cultured cells were lysed in RIPA buffer (1% Nonidet P-40, 50 mM Tris–HCl [pH 7.4], 150 mM NaCl, 5 mM EDTA, 0.1% SDS, 1% sodium deoxycholate) supplemented with protease and phosphatase inhibitor cocktail (Nacalai Tesque, Inc., Kyoto, Japan). The lysate was centrifuged at 12,000 rpm at 4 °C for 20 min, and the supernatant was collected. Equal amount of lysates were separated by 4–15% SDS-PAGE (Bio-Rad, Hercules, CA, USA) and transferred to a polyvinylidene fluoride membrane (Millipore, Billerica, MA, USA). The membrane was incubated in polyvinylidene blocking reagent (Toyobo, Tokyo, Japan) at room temperature for 20 min, and then incubated at 4 °C overnight with the appropriate primary antibody diluted in Can Get Signal Solution 1 (Toyobo). The primary antibody was detected by a horseradish peroxidase–conjugated secondary antibody diluted in Tris-buffered saline (pH 7.4) containing 0.1% Tween-20, and visualized with Luminata Forte Western HRP substrate (Millipore, Billerica, MA, USA). An image of the membrane was acquired on a LAS-4000 mini luminescent image analyzer (Fujifilm, Tokyo, Japan). The primary antibodies used for the analysis were as follows: anti-phospho-VASP (Ser239) (#3114, Cell Signaling Technology, Beverly, MA, USA), anti-phospho-VASP (Ser157) antibody (#3111, Cell Signaling Technology), anti-VASP (#3112, Cell Signaling Technology), anti-phospho-Smad1 (Ser463/465) / Smad5 (Ser463/465) / Smad8 (Ser426/428) (#9511, Cell Signaling Technology), rabbit anti-Smad1 mAb (#6944, Cell Signaling Technology), rabbit anti-phospho-Smad2 mAb (#3108, Cell Signaling Technology), mouse anti-Smad2 mAb (#3103, Cell Signaling Technology), and mouse anti-GAPDH mAb (sc-32233, Santa Cruz Biotech, Dallas, TX, USA).

### Statistical analysis

Data are expressed as means ± SEM. Between-group comparisons were performed using the unpaired Student’s *t*-test. For multiple-group comparisons, one-way ANOVA, followed by the post-hoc Fisher’s least significant difference test, was used. *P* < 0.05 was considered to be significant.

## Results

### ANP attenuated BLM-induced pulmonary fibrosis and inflammation in mice

**Fig. 1:**
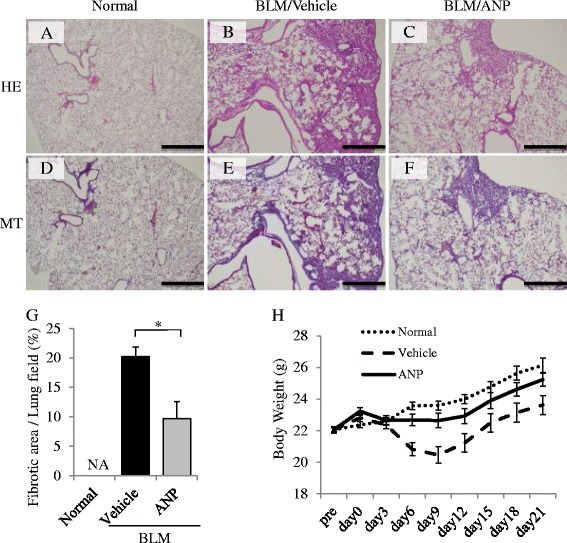
ANP attenuates BLM-induced pulmonary fibrosis in mice. BLM was administered intratracheally to C57BL/6 mice on Day 0, and samples were removed on Day 21. ANP or vehicle was subcutaneously infused using an osmotic mini-pump throughout the experiment. Representative micrographs of lung tissue stained with hematoxylin–eosin (HE: *upper panels*) and Masson trichrome (MT: *lower panels*): normal control mice (**a**, **d**), BLM-treated mice receiving vehicle (**b**, **e**), and BLM-treated mice receiving ANP (**c**, **f**). Scale bar: 500 μm. **g** Fibrotic area, measured using image analysis software, and is expressed as a percentage of the whole lung field. Values represent means ± SEMs (*n* = 5 mice per group). *p < 0.05. NA: not assessed because of the absence of fibrotic area in normal lungs. **h** Body weight changes of mice after BLM administration. Values represent means ± SEMs (in normal control group, *n* = 13; in BLM with vehicle group, *n* = 15, in BLM with ANP group, *n* = 14)

**Fig. 2:**
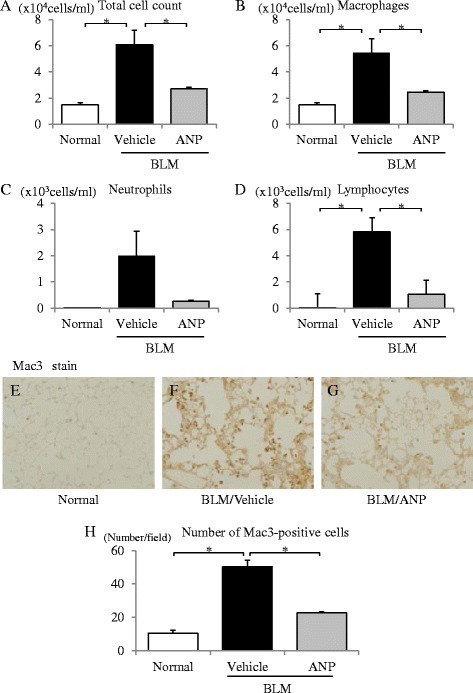
ANP attenuates inflammation in the lungs induced by BLM administration. Numbers of total cells (**a**), macrophages (**b**), neutrophils (**c**), and lymphocytes (**d**) in bronchoalveolar lavage (BAL) fluid on Day 7 after BLM administration. Values represent means ± SEMs (*n* = 5 mice per group). *p < 0.05. Lung sections from normal control mice (**e**), BLM-treated mice receiving vehicle (**f**), and BLM-treated mice receiving ANP treatment (**g**) obtained 7 days after BLM administration were stained with Mac-3. Representative images are shown at 200× magnification. **h** Mac-3–positive cells were counted in ten high-power fields (HPF). The data are expressed as means ± SE (in normal control group, *n* = 4; in the other groups, *n* = 5. *p < 0.05)

First, we examined the in vivo anti-fibrotic and anti-inflammatory effects of ANP on BLM-induced pulmonary fibrosis in mice. Histological examination of the lungs in mice after BLM administration revealed lung parenchymal fibrotic changes in comparison with the normal control group (Fig. 1a, b, d, e). Compared with vehicle, ANP pretreatment significantly attenuated BLM-induced lung fibrotic changes (Fig. 1a–f). Quantitative assessment of the severity of lung fibrosis in MT-stained tissue sections demonstrated that ANP pretreatment significantly attenuated BLM-induced lung fibrotic lesions relative to vehicle (Fig. 1g). More than 3 days after BLM administration, significant body weight loss was observed in the vehicle-treated group relative to the normal control group. However, ANP pretreatment significantly attenuated BLM-induced weight loss (Fig. 1h). To investigate the accumulation of inflammatory cells in the lungs, we examined the number of inflammatory cells in BAL fluid and performed Mac3 staining of the lung. In BAL fluid, both total and individual cell counts were significantly elevated in the vehicle-treated group relative to the normal control group. ANP pretreatment significantly decreased total, macrophage, and lymphocyte cell counts relative to vehicle (Fig. 2a-d). Quantitative assessment of the number of inflamed cells, as determined by Mac3 staining, demonstrated that ANP pretreatment significantly attenuated the number of inflammatory cells in the lungs induced by BLM relative to vehicle (Fig. 2e-h). These results indicate that ANP can attenuate the fibrotic changes and accumulation of inflammatory cells in BLM-induced pulmonary fibrosis.

### ANP attenuated the expression of cytokines induced by BLM in mouse lung

**Fig. 3:**
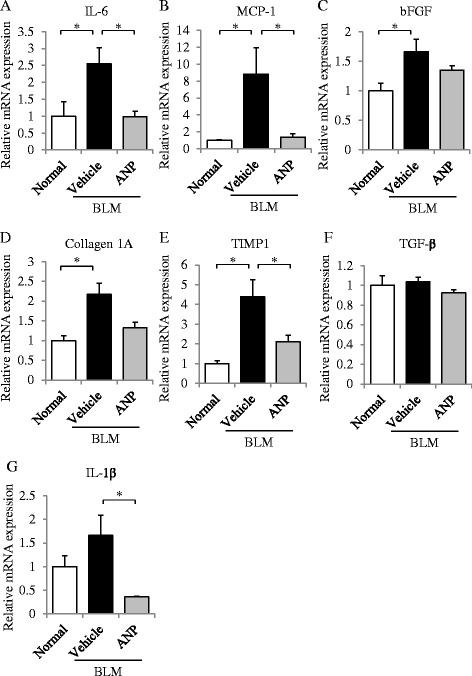
ANP attenuates the elevated mRNA levels of pro-inflammatory and pro-fibrotic cytokines in BLM-administered mice. Quantitative RT-PCR analysis of IL-6 (**a**), MCP-1 (**b**), bFGF (**c**), collagen 1A (**d**), TIMP1 (**e**), TGF-β (**f**), and IL-1β (**g**) in lung tissues 21 days after BLM administration. Relative mRNA level of each cytokine in normal control or BLM/vehicle-treated or BLM/ANP-treated mice are shown. Values represent means ± SEMs (*n* = 5 mice per group). *p < 0.05

To evaluate the anti-inflammatory and anti-fibrotic effects of ANP in BLM-induced lung fibrosis, we analyzed mRNA expression changes of pro-inflammatory cytokines and pro-fibrotic cytokines in the lungs. With the exception of TGF-β and IL-1β, expression levels of several mRNAs were significantly elevated in the vehicle-treated group after BLM administration in comparison with the normal control group. ANP pretreatment significantly reduced the expression levels of IL-6, MCP-1, TIMP1, and IL-1β relative to vehicle (Fig. 3a, b, e, g). The gene expression levels of bFGF and collagen 1A were lower in the ANP-treated group than in the vehicle-treated group, but the difference was not significant (Fig. 3c, d). By contrast, the mRNA level of TGF-β was not significantly altered by BLM or ANP treatment (Fig. 3f). These results indicate that ANP has the potential to reduce the production of pro-inflammatory and pro-fibrotic cytokines and collagen accumulation associated with pulmonary fibrosis in mice.

### Anti-fibrotic and inflammatory effects of vascular endothelium–specific *GC-A* overexpressed mice in BLM-induced pulmonary fibrosis

**Fig. 4:**
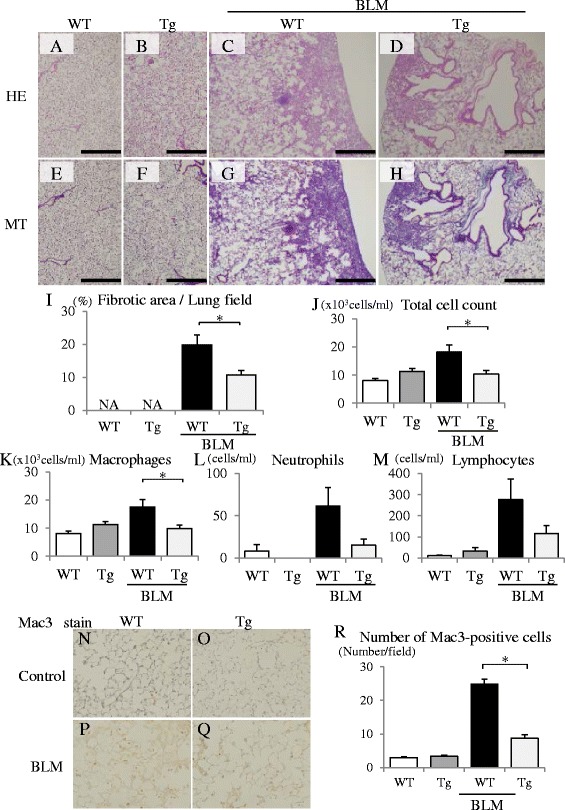
Anti-fibrotic and inflammatory effects of vascular endothelium–specific *GC-A* overexpressed mice in BLM-induced pulmonary fibrosis. BLM was administered intratracheally into vascular endothelium–specific *GC-A* overexpressed (Tg) mice and WT littermates on Day 0, and lung tissues were removed on Day 21. Representative micrographs of lung tissue stained with hematoxylin–eosin (HE: *upper panels*) and Masson trichrome (MT: *lower panels*): WT littermates treated without BLM (**a**, **e**), vascular endothelium–specific *GC-A* overexpressed mice treated without BLM (**b**, **f**), WT littermates treated with BLM (**c**, **g**), and vascular endothelium–specific *GC-A* overexpressed mice treated with BLM (**d**, **h**) are shown. Scale bar: 500 μm. **i** Fibrotic area was measured using image analysis software and is expressed as a percentage of the whole lung field. Values represent means ± SEM (WT without BLM, *n* = 4; Tg without BLM, *n* = 3; WT with BLM, *n* = 5; Tg with BLM, *n* = 5). *p < 0.05. NA, not assessed because of the absence of fibrotic area in normal lungs. **j**–**m** Numbers of total cells (**j**), macrophages (**k**), neutrophils (**l**), and lymphocytes (**m**) in bronchoalveolar lavage (BAL) fluid on Day 21 after BLM administration. Values represent means ± SEM (*n* = 5 mice per group). *p < 0.05. Lung sections obtained 21 days after BLM administration were stained with Mac-3 in WT littermates treated without BLM (**n**), vascular endothelium–specific *GC-A* overexpressed mice treated without BLM (**o**), WT littermates treated with BLM (**p**), and vascular endothelium-specific *GC-A* overexpressed mice treated with BLM (**q**). Representative images are shown at 200× magnification. Macrophages were identified by Mac-3 staining. **r** Mac-3–positive cells were counted in ten high-power fields (HPF), and the data are expressed as means ± SE. (*n* = 3–5 mice per group). *p < 0.05

Because vascular endothelial cells play a major role of BLM-induced pulmonary fibrosis, we hypothesized that GC-A expressed on vascular endothelial cells might be responsible for the anti-fibrotic and inflammatory effects of ANP. Accordingly, to determine the target of the anti-fibrotic and inflammatory effects of ANP in BLM-induced pulmonary fibrosis, we used vascular endothelium–specific *GC-A* overexpressed mice. BLM-induced pulmonary fibrosis was reduced in vascular endothelium–specific *GC-A* overexpressed mice relative to WT littermates (Fig. 4a-h). Quantitative histological analysis revealed that BLM-induced fibrotic changes were significantly reduced in vascular endothelium–specific *GC-A* overexpressed mice (Fig. 4i). In BAL fluid, total and macrophages cell counts were significantly reduced in the overexpressed mice (Fig. 4j, k). Cell counts of neutrophils and lymphocytes were also lower in the overexpressed mice, but the difference was not significant (Fig. 4l, m). Furthermore quantitative assessment of the number of inflamed cells in Mac3 staining confirmed that the number of inflammatory cells in the lung was significantly lower in the overexpressed mice (Fig. 4n-r). These results indicate that ANP exerts its anti-fibrotic and inflammatory effects in the lung via vascular endothelial cells.

### Effects of ANP on mouse endothelial cells in TGF-β signaling

**Fig. 5:**
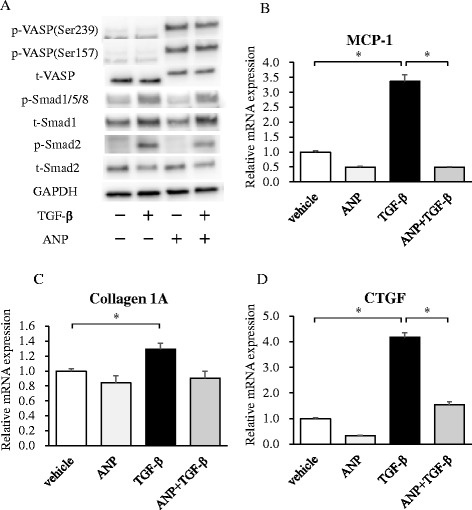
Effects of ANP on TGF-β signaling in mouse endothelial cells. **a** Western blot analysis with the antibodies indicated at left using the cell lysates prepared from SVEC/GC-A cells stimulated with TGF-β (1 ng/ml) and/or ANP (0.1 μM) for 30 min. The blot shown is representative of three independent experiments. **b**–**d** Quantitative RT-PCR analysis of MCP-1 (**b**), collagen 1A (**c**), and CTGF (**d**) in SVEC/GC-A stimulated with TGF-β (1 ng/ml) and/or ANP (0.1 μM) for 4 h. Relative mRNA expressions in vehicle, ANP, TGF-β/vehicle, and TGF-β/ANP groups are shown. Values represent means ± SEMs (*n* = 6 per group). *p < 0.05

To investigate the molecular mechanism of ANP/GC-A signaling in endothelial cells, we established a mouse immortalized endothelial cell line stably expressing GC-A (SVEC/GC-A). First, we observed robust phosphorylation of VASP at Ser157 and Ser239 in SVEC/GC-A cells following ANP administration, indicating that stable expression of GC-A confers a high level of responsiveness to ANP (Fig. 5a). Second, to examine the effects of ANP on endothelial cells, we analyzed protein expression, phosphorylation levels, and mRNA expression in SVEC/GC-A stimulated with TGF-β and/or ANP. In response to TGF-β, phosphorylation of Smad2 was elevated in SVEC/GC-A cells (Fig. 5a). ANP pretreatment decreased phosphorylation of Smad2 after TGF-β stimulation (Fig. 5a). The phosphorylation levels of Smad1/5/8 were similar between ANP-treated and vehicle-treated SVEC/GC-A cells. In addition, mRNA levels of MCP-1, collagen 1A, and CTGF were significantly elevated after TGF-β stimulation in SVEC/GC-A cells (Fig. 5b-d). ANP significantly decreased the expression levels of MCP-1 and CTGF following TGF-β stimulation (Fig. 5b, d). The mRNA level of collagen 1A was lower in ANP-treated SVEC/GC-A cells, however the difference was not significant (Fig. 5c). These results suggest that ANP attenuates the TGF-β/Smad2 signaling pathway in endothelial cells.

## Discussion

By comparing ANP-treated mice with vehicle mice, and vascular endothelium–specific *GC-A* overexpressed with WT mice, in a BLM-induced pulmonary fibrosis model, we showed for the first time that ANP exerts an anti-fibrotic effect on BLM-induced pulmonary fibrosis via vascular endothelial cells. Our findings indicate that ANP treatment represents a promising option for preventing the progression of pulmonary fibrosis.

Previous studies showed that pulmonary inflammation is responsible for pulmonary fibrosis in humans and BLM-treated mice [4, 5, 16]. Several proinflammatory cytokines, including MCP-1 and IL-6, are involved in pulmonary inflammation and the development of BLM-induced pulmonary fibrosis in mice [17–19]. A previous study showed that anti-MCP-1 therapy using gene transfection could reduce BLM-induced pulmonary fibrosis in mice [19]. In this study, ANP significantly attenuated the elevated mRNA levels of MCP-1and IL-6 in BLM-induced pulmonary fibrosis.

Activated endothelial cells secrete proinflammatory cytokines and profibrotic mediators, which recruit and activate inflammatory cells and fibroblasts, resulting in collagen deposition [4, 5]. A previous study showed that vascular endothelial cells exposed to BLM in vitro increased the secretion of certain profibrotic mediators [20]. In addition, activated endothelial cells can contribute to prolonged tissue injury by promoting the expression of proinflammatory cytokines and adhesion molecules, resulting in leukocyte homing and the extravasation of cells at sites of inflammation [4, 5]. However, little is known about the benefits of targeting vascular endothelial cells in BLM-induced pulmonary fibrosis in vivo. In this study, we showed that ANP acts on vascular endothelial cells, resulting in anti-fibrotic and inflammatory effects, in BLM-induced pulmonary fibrosis.

TGF-β is a key signaling molecule involved in the initiation and enhancement of tissue fibrosis [21]. Previous studies showed that the development of fibrosis in various tissues including the lung is less severe in Smad3-deficient mice than in control mice [22, 23]. Therefore, the TGF-β/Smad3 pathway plays a key role in the mechanisms leading to fibrosis following tissue injury. Previous studies showed that ANP/cGMP signaling inhibits TGF-β–induced Smad2 and Smad3 nuclear translocation in pulmonary arterial smooth muscle cells [24, 25]. However, little is known about the effects of ANP on vascular endothelial cells in TGF-β signaling. In this study, we found that ANP attenuated pulmonary fibrosis and inflammation induced by BLM, at least in part, via inhibition of Smad2 phosphorylation in TGF-β signaling. However, we could not determine the detailed mechanisms underlying the effects of ANP treatments on TGF-β signaling. Recent studies showed that the most obvious involvement of endothelial cells is related to endothelial-to-mesenchymal transition (EndoMT) in the context of pulmonary diseases such as pulmonary hypertension or pulmonary fibrosis [26, 27]. In addition, we could not investigate the effects of ANP on functional investigations including bronchoconstriction in this study. Therefore, further studies are required to elucidate these issues.

ANP is an endogenous peptide that has been approved in Japan for treatment of acute heart failure since 1995, and adverse events from its use are very rare. Therefore, the clinical safety of ANP has already been established. Recently, we reported that ANP not only had prophylactic effects on postoperative cardiopulmonary complications, but also exerted protective effect from postoperative cancer recurrence after surgical resection of tumors [12–14]. We showed that ANP prevents cancer metastasis via vascular endothelial cells [14]. Therefore, ANP represents a promising option for the safe treatment of various diseases.

## Conclusions

In conclusion, this study is the first to show that ANP exerts anti-fibrotic and anti-inflammatory effects in BLM-induced pulmonary fibrosis via vascular endothelial cells, possibly by attenuating the phosphorylation of Smad2 in TGF-β signaling.

## Abbreviations

ANP: Atrial natriuretic peptide
GC-A: Guanylyl cyclase-A
BLM: Bleomycin
TGF-β: Transforming growth factor-β
WT: Wild-type
BAL: Bronchoalveolar lavage
MT: Masson trichrome
PBS: Phosphate-buffered saline
IL: Interleukin
bFGF: Basic fibroblast growth factor
CTGF: Connective tissue growth factor
MCP-1: Monocyte chemoattractant protein-1
TIMP1: Tissue inhibitor of metalloproteinases type1
DMEM: Dulbecco's Modified Eagle Medium
FCS: Fetal calf serum
SVEC: A mouse immortalized endothelial cell line
SVEC/GC-A: SVEC cells stably expressing GC-A-FLAG.
